# Recurrent Rhabdomyolysis in a Medical Cadet during Military Training as a Rare Initial Presentation in Calpainopathy

**DOI:** 10.1155/2024/2775517

**Published:** 2024-09-02

**Authors:** Sethapong Lertsakulbunlue, Boonsub Sakboonyarat, Piradee Suwanpakdee, Boonchai Boonyawat

**Affiliations:** ^1^ Department of Pharmacology Phramongkutklao College of Medicine, Bangkok 10400, Thailand; ^2^ Department of Military and Community Medicine Phramongkutklao College of Medicine, Bangkok 10400, Thailand; ^3^ Division of Neurology Department of Pediatrics Phramongkutklao Hospital and Phramongkutklao College of Medicine, Bangkok 10400, Thailand; ^4^ Division of Medical Genetics Department of Pediatrics Phramongkutklao Hospital and Phramongkutklao College of Medicine, Bangkok 10400, Thailand

## Abstract

Rhabdomyolysis, an emergency medical condition linked to muscle necrosis and intracellular substances released into the bloodstream, significantly endangers military personnel in heat-stress conditions. Rhabdomyolysis can also be an initial presentation in inherited muscle disorders. This study reports a novel case of calpainopathy (LGMDR1) diagnosed in a 19-year-old male military cadet who initially presented with recurrent rhabdomyolysis during training, a rare presentation in LGMD patients. Furthermore, a persistent creatine kinase (CK) elevation was observed at baseline. The diagnosis was confirmed by identifying a compound heterozygous of a novel frameshift, c.606dup (p.Ala203CysfsTer9), a mutation in exon 4, and a missense, c.956C > T (p.Pro319Leu), a mutation in exon 7 of the *CAPN3* gene, via whole exome sequencing. This case highlights the necessity of diagnostic investigation in individuals who have persistent high CK levels during the rhabdomyolysis episodes and possibly CK screening prior to military training to preemptively identify and mitigate complications from undiagnosed muscular dystrophies in military personnel in the future.

## 1. Introduction

Rhabdomyolysis constitutes a clinical emergency characterized by extensive muscle necrosis and consequent release of intracellular muscle components into circulation [1]. This condition poses a significant threat among military personnel, particularly those exercising under severe physical effort and heat stress [2]. Nevertheless, the classical triad of symptoms—muscle aches, weakness, and dark urine—manifests in less than 10% of patients, leading to diagnostic delays [3, 4]. Furthermore, exertional rhabdomyolysis can be an initial indicator of underlying genetic muscle disorders that reduce the exercise threshold for muscle breakdown [3]. Although exertional rhabdomyolysis is typical in many metabolic myopathies, it may also be the initial presentation in some muscular dystrophies, including dystrophinopathy, *FKRP*-muscular dystrophy (LGMD2I), anoctaminopathy (LGMD2L), and dysferlinopathy (LGMD2B) [4, 5]. Exertional rhabdomyolysis is a rare presentation in calpainopathy and has been reported in only two cases [4]. Herein, we present a case of a 19-year-old male medical cadet who exhibited recurrent rhabdomyolysis while undergoing military training. Genetic analysis revealed compound heterozygous mutations in the *CAPN3* gene, making a first-time diagnosis of calpainopathy (LGMDR1) in our patient.

## 2. Case Report

A 19-year-old male medical cadet presented with myalgia, proximal muscle weakness, and dark urine during intense military training. He was diagnosed with exertional rhabdomyolysis triggered by heat injury and heavy exercise. A history of bilateral proximal lower limb pain with no muscle weakness has been reported for a year. There was no history of breathlessness, sensory loss, cranial nerve involvement, or bowel-bladder dysfunction. Additionally, there was no history of illicit drug use. He is the only child of healthy, non-consanguineous parents. He was previously in good health. Physical examination revealed mild waddling gait and bilateral calf pseudo-hypertrophy. Muscle strength was assessed, and proximal muscle weakness was revealed with a positive Gowers's sign. Other neurological examinations were unremarkable.

Laboratory investigation confirmed a very high serum creatine kinase (CK) of 401,293 U/L (normal: 25–200). The patient's initial biochemistry laboratory results are shown in Table 1. Elevation of aspartate aminotransferase, alanine aminotransferase, lactate dehydrogenase, and uric acid was observed. Urinalysis demonstrated a yellow/turbid appearance with a specific gravity of 1.050, protein (3+), and erythrocytes (4+). The complete blood count and thyroid function tests were unremarkable. Electrocardiogram and echocardiography revealed no cardiac involvement.

Furthermore, our patient still had two subsequent episodes of exertional rhabdomyolysis despite a reduction in a regular training routine. Consequently, he has been excluded from military training to focus on recuperation and preparing for medical studies. He presented with myalgia and dark urine accompanied by a CK level exceeding 100,000 U/L in both episodes. During the six-month follow-up period, serum CK levels remained consistently elevated at 7,000–10,000 U/L. To monitor for the possibility of kidney injury, serum creatinine was also followed up and revealed normal results. Consultation with a neurologist and medical geneticist was executed to identify the underlying cause of persistent CK elevations and recurrent rhabdomyolysis. Further investigations were needed. Electromyography (EMG) was performed on the gluteus medius and gastrocnemius muscles and revealed electrophysiological evidence consistent with a myopathic pattern. Histopathologic evaluation of a gastrocnemius muscle biopsy exhibited variation in muscle fiber size, an increase in central nuclei, and endomysial fibrosis, consistent with myopathy, dystrophic process. Muscular dystrophy was suggested. Unfortunately, an immunohistochemical stain for the specified cause of muscular dystrophy was unavailable in our institution. Thus, whole exome sequencing is the method of choice for identifying our patient's genetic cause of recurrent rhabdomyolysis.

### 2.1. Molecular Methods and Results

Blood samples were collected from our patient and his mother. Genomic DNA was extracted using the QIAmp DNA Blood Mini Kit (Qiagen, Germany). Whole exome sequencing was performed. The exome library was prepared using SureSelect Human All Exon V7 (Agilent). All sequencing was performed on the NovaSeq 6000 platform (Illumina) (Macrogen, Korea). Alignment and variant calling were performed by BWA and GATK, respectively.

**Figure 1:**
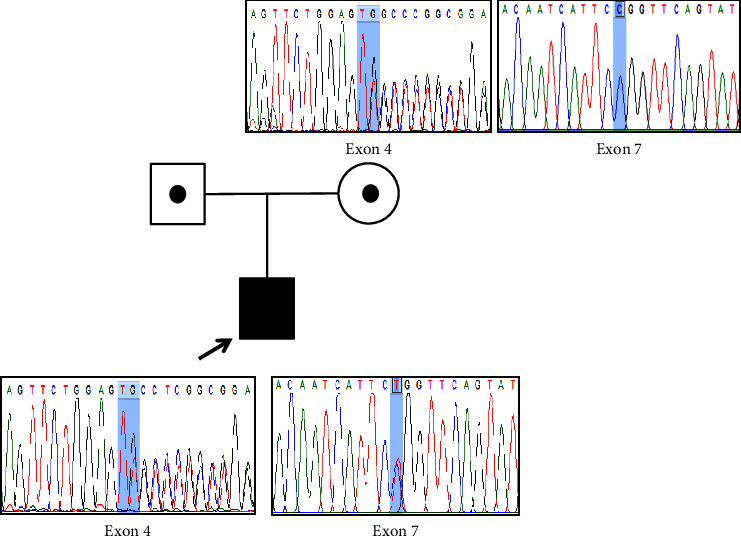
Sanger sequencing of the patient's DNA showed compound heterozygous c.606dup mutation in exon 4 and heterozygous c.956C > T mutation in exon 7 of the *CAPN3* gene. The former c.606dup mutation was inherited from the maternal DNA.

After variant analysis, a compound heterozygous of a novel frameshift mutation, c.606dup (p.Ala203CysfsTer9), in exon 4 and a previously reported missense mutation, c.956C > T (p.Pro319Leu), in exon 7 of *CAPN3* (NM_000070.3) gene were identified. Other genetic causes of muscular dystrophies and LGMDs were also excluded by WES analysis. Sanger sequencing revealed that the former frameshift mutation was inherited from his mother, and the inheritance of the latter missense mutation could not be confirmed due to the unavailability of the paternal DNA (Figure 1). However, this suggested that these mutations were biallelic. According to the ACMG classification in 2015 [6], both mutations were predicted to be pathogenic. The PVS (null variant), PM2 (absent in population database), and PM3 (in *trans* with a pathogenic variant) criteria were used for c.606dup mutation and PM1 (mutational hot spot), PM2, PM3, PP3 (computational evidences support a deleterious effect), and PP5 (reputable source reports pathogenic) criteria were used for c.956C > T mutation.

## 3. Discussion

Rhabdomyolysis is a critical condition characterized by a substantial increase in serum CK levels. Various rhabdomyolysis etiologies, including acquired and inherited causes, are identified [5]. Recurrent rhabdomyolysis and persistent elevation of serum CK levels may serve as an initial indicator for inherited muscle disorders that reduce the exercise threshold for muscle breakdown [3, 5]. Thus, exercise is one of the most common triggers for rhabdomyolysis in these patients. The association between rhabdomyolysis and muscular dystrophy is not well recognized [4, 5].

In this report, we describe an atypical manifestation of calpainopathy presenting with recurrent rhabdomyolysis at the onset of disease and persistently elevated CK levels during military training in an adolescent male medical cadet. Rhabdomyolysis was previously reported in only two cases of calpainopathy [4]. The first patient was a 25-year-old man presenting with multiple episodes of exercise-induced rhabdomyolysis with no reported CK level at the time of the attacks. The second patient was a 48-year-old man presenting with an episode of rhabdomyolysis triggered by exercise with a CK level of 186,000 IU/L. Proximal muscle weakness was detected in both patients. The baseline CK levels of both patients were 1,000 and 1,300 IU/L, respectively. Genetic analysis of the *CAPN3* gene revealed a compound heterozygous of an inframe deletion: c.759_761del (p.Lys754del), and a missense mutation: c.551C > T (p.Thr184Met), in the first patient and a compound heterozygous of two missense mutations: c.1505T > C (p.Ile502Thr) and c.1327T > C (p.Ser443pro), in the second patient. As in our patient, who was previously healthy, intensive exercise during military training is the trigger of recurrent rhabdomyolysis. Thus, heat injury is our patient's first suspected cause of rhabdomyolysis. However, persistent elevation of baseline CK levels during the episodes is the most important clue for inherited muscle disorders. Whole exome sequencing was the most helpful investigation for making a specific diagnosis of calpainopathy in our patient. A novel heterozygous frameshift mutation: c.606dup (p.Ala203CysfsTer9), in exon 4 and a previously reported missense mutation: c.956C > T (p.Pro319Leu), in exon 7 of *CAPN3* gene were identified in our patient DNA.

Calpainopathy is the most prevalent form of LGMDs and is caused by mutations in the *CAPN3* gene [7]. This gene encodes an 821-amino acid CAPN3 muscle-specific protein involved in myofibrillogenesis and sarcomere remodeling. CAPN3 is a calcium-activated heterodimer protease and is composed of several domains, including a cysteine protease domain (PC1 and PC2), a Calpain-type Beta-SandWich (CBSW) domain, and a Penta E-F hand (PEF) domain [8, 9]. The novel c.606dup frameshift mutation produces a truncated protein of only 211 residues, which causes elimination of all parts of the PEF, CBSW, and PC2 domains and some parts of the PC1 domain. This truncated protein will be removed from the cell by nonsense-mediated decay, resulting in the loss of CAPN3 protein. c.956C > T (p.Pro319Leu) has been previously reported in two patients [10, 11]. Both patients were first diagnosed at 15 and 23 years of age, and they were still ambulant at the age of 32 and 58 years, respectively. This mutation was in compound heterozygous with c.257C > T (p.Ser86Phe) mutation in the first patient and c.2257delGinsAA (p.Asp753LysfsTer12) mutation in the second patient, respectively. This suggested that c.956C > T mutation was presented only in LGMD patients with mild phenotypes. Neither of them presented with rhabdomyolysis.

Calpainopathy is mainly characterized by symmetrical and progressive proximal muscle weakness. The clinical course is highly variable in the age of onset and severity of the disease [12, 13]. Exertional rhabdomyolysis is an unusual manifestation of calpainopathy. Until now, more than 520 mutations in the *CAPN3* gene have been reported in both the Leiden Open Variation Database (LOVD) and the Human Gene Mutation Database (HGMD). Although the genotype-phenotype correlation in calpainopathy is not fully demonstrated, most cases with either homozygous or compound heterozygous null alleles, including nonsense, frameshift, and splice-site mutations, are frequently associated with the most severe phenotypes of the disease [10, 12, 13]. Rhabdomyolysis is presented in only calpainopathy individuals whose one of either mutated alleles is a missense mutation, as shown in our case and two previously reported patients [4]. This indicates that exertional rhabdomyolysis can be one of the atypical manifestations in calpainopathy with mild to moderate severity.

Elevated CK levels have been shown to indicate a risk of muscle disorders. Therefore, implementing CK screening for individuals engaged in activities that require physical exertion, such as military training, may be beneficial. A systematic review demonstrated the good accuracy of the CK test in screening for Duchenne muscular dystrophy among newborns [14]. To the best of our knowledge, a protocol for screening muscular dystrophy before military training has never been reported. Implementing CK screening for the early detection of inherited muscle disorders, including muscular dystrophy, could further enhance rhabdomyolysis prevention. This approach would allow us to identify individuals who may benefit from subsequent genetic testing to elucidate the nature of rhabdomyolysis.

## 4. Conclusion

We described an adolescent male medical cadet who presented with recurrent rhabdomyolysis during military training. Calpainopathy was diagnosed after a biallelic *CAPN3* mutation was identified by whole exome sequencing. This case highlights the importance of diagnostic investigation of inherited muscle disorders in individuals who have persistent elevation of CK levels during rhabdomyolysis episodes. Therefore, rhabdomyolysis screening by implementing CK levels and developing a comprehensive protocol for clinical examinations could be beneficial before military training.

## Figures and Tables

**Table 1:** Patient's initial biochemistry laboratory results from the first visit.

Laboratory test	Result	Normal level
Serum creatine kinase (U/L)	**401,293**	25–200
Liver function tests		
Total protein (g/dL)	6.8	6–8.5
Albumin (g/dL)	4.2	2.6–5.2
Total bilirubin (mg/dL)	1	0-1
Direct bilirubin (mg/dL)	0.3	0–0.4
Aspartate aminotransferase (U/L)	**4,166**	10–40
Alanine aminotransferase (U/L)	**1,515**	7–56
Alkaline phosphatase (U/L)	43	40–129
Lactate dehydrogenase (U/L)	**579**	135–225
Uric acid (*μ*mol/L)	**797**	240–510
Serum blood urea nitrogen (mg/dL)	**23.6**	6–20
Serum creatinine (mg/dL)	1	0.67–1.17
Serum electrolytes		
Sodium (mEq/L)	135	135–145
Potassium (mEq/L)	4.1	3.5–5
Chloride (mEq/L)	99	98–110
Bicarbonate (mEq/L)	27	21–31

Bold indicates out of normal range result.

## Data Availability

The data used to support the findings of this study are available from the corresponding author upon reasonable request.
